# Some Socioeconomic Factors and Lifestyle Habits Influencing the Prevalence of Obesity among Adolescent Male Students in the Hail Region of Saudi Arabia

**DOI:** 10.3390/children5030039

**Published:** 2018-03-09

**Authors:** Awfa Y. Alazzeh, Eyad M. AlShammari, Majdi M. Smadi, Firas S. Azzeh, Bandar T. AlShammari, Suneetha Epuru, Shahidah Banu, Rafia Bano, Shadi Sulaiman, Jerold C. Alcantara, Syed A. Ashraf, Samir Qiblawi

**Affiliations:** 1 Department of Clinical Nutrition, College of Applied Medical Sciences, University of Hail, P.O. Box 2440, Hail 55462, Saudi Arabia; eyadhealth@hotmail.com (E.M.A.); majdi75s@yahoo.com (M.M.S.); ba108068398@gmail.com (B.T.A.); epurusuneetha@gmail.com (S.E.); shahidaed1@gmail.com (S.B.); rafiazafar78@gmail.com (R.B.); amirashrafy2007@gmail.com (S.A.A.); 2 Department of Clinical Nutrition, Faculty of Applied Medical Sciences, Umm Al-Qura University, Makkah 24231, Saudi Arabia; fsazzeh@uqu.edu.sa; 3 Department of Clinical Laboratory Sciences, Faculty of Applied Medical Sciences, University of Hail, Hail 55462, Saudi Arabia; shadisolyman@hotmail.com (S.S.); j.alcantara@uoh.edu.sa (J.C.A.); 4 Department of Histopathology, Faculty of Medicine, University of Hail, Hail 55462, Saudi Arabia; drsam_2003@yahoo.com

**Keywords:** obesity, hail region, socioeconomic factors, lifestyle habits, male adolescents

## Abstract

A cross-sectional study was conducted to investigate the effect of some socioeconomic factors and lifestyle habits on the prevalence of obesity among adolescent male students in the Hail region, Saudi Arabia. A questionnaire was filled by 1495 male adolescents distributed among 12 schools in the Hail region. Body weight and height were taken, and the *Z*-score of students was measured using Anthroplus software with a cutoff 1–2 and +2 standard deviations to determine overweight and obesity, respectively. The study revealed that 21.3% of students were overweight and 27% were obese, respectively. There was a negative association between family size of >8 and obesity (OR: 0.68, CI: 0.48–0.92, *p* = 0.05). Family income of <5000 SR was negatively associated with obesity (OR: 0.59, CI: 0.36–0.97, *p* = 0.03). Whether a subject’s mother worked (odds ratio (OR): 1.43, confidence interval CI: 1.03–1.99, *p* = 0.03) as well as the subject’s mother’s education—whether she can read and write, has a middle school degree, or has done postsecondary studies—were positively associated with obesity. Exercise, regardless of the duration, was negatively associated with obesity. In addition, sleeping <6 h/day had a positive association with obesity. Conclusion: a >8 family size and a low family income were negatively associated with obesity, while having an educated and working mother was positively associated with obesity.

## 1. Introduction

There is an increasing awareness to the dangers of obesity on health status, life quality, and life expectancy. Many studies have focused on the etiology of obesity, protective means, and ways to prevent its incidence. Some reviews have discussed that in detail [1,2,3]. Health effects of childhood and adolescent obesity are critically important due to their consequences on the health status in adult ages. Childhood and adolescent obesity was shown to significantly increase adult obesity along with major health risks such as type 2 diabetes, increased incidence of metabolic syndrome, and different types of cancers [4].

Studies on obesity in children and adolescents among different regions of Saudi Arabia revealed that 12% of participating male children in the northern region of Saudi Arabia, which Hail is a part of, were overweight, and 7% were obese [5]. Another study showed that the prevalence of overweight in adolescent males in the Riyadh region of Saudi Arabia was 13.8% and obesity was 20.5% [6]. However, in the Abha city of the southern region of Saudi Arabia, only 5% of adolescent students were overweight and 11% were obese [7]. In adult ages, studies have shown that the Hail region had the highest percentage of obese adults among other regions of Saudi Arabia as more than 33.9% of adults living in the Hail region are suffering from obesity [8]. A more recent study by Ahmed et al. [9] revealed that the prevalence of obesity in the Hail region among adults who attend primary health care facilities was 63.6%, (56.2% and 71.0% for males and females, respectively). This emphasizes the contribution of lifestyle and socioeconomic factors during early ages in the prevalence of obesity.

A study by Al-Nuaim et al. [10] showed that lifestyle factors would affect the prevalence of obesity among students in the eastern region of Saudi Arabia. Al-Hazzaa et al. [11] showed that the irregular sleep pattern would increase the prevalence of obesity among adolescent students in Saudi Arabia. Another study by Al-Ghamdi [12] revealed that watching TV is associated with the prevalence of obesity among schoolchildren in the Riyadh region. These previous studies showed the importance of studying the effect of socioeconomic factors and lifestyle habits on the prevalence of obesity. 

In previous decades, the Hail region showed a shift from an agriculture-based economy toward more urbanization. This resulted in a change in lifestyle habits and a replacement of traditional food with Western-style fast food. Since adults in the Hail region have the highest percentage of obesity in the Kingdom of Saudi Arabia (KSA), which might be a result of childhood practices and socioeconomic effects, it is critical to understand which risk factors increase the prevalence of obesity in the Hail region during adolescence. This is crucial for intervention programs to be successful, since obesity is considered a preventable disease [13].

## 2. Materials and Methods

### 2.1. Subjects and Study Design

A cross-sectional study was conducted from January–May 2016 to investigate the effect of some socioeconomic factors and lifestyle habits on the prevalence of obesity among adolescent male students. The inclusion criteria were Saudi male adolescents 12–19 years of age attending middle and high schools in the Hail region, Kingdom of Saudi Arabia. The exclusion criteria were students who suffered from chronic diseases, such as diabetes or any heart, kidney, and anemic disease. Multi-stage sampling was used to randomly select participants in the schools of the Hail region. Twelve male middle and high schools were selected. The original number of participants was 1638 students; however, only 1495 male students were enrolled after cleaning the data and removing incomplete questionnaires with missing data (*n* = 26). Sociodemographic characteristics of the study group are shown in Table 1.

### 2.2. Data Collection

A structured and validated questionnaire was introduced to the participants. Students were asked, with the help of trained dieticians and technicians, to answer questions about their socioeconomic status and lifestyle habits. The objectives of the study were sent to the parents, and written consent forms were obtained from them before the onset of the study. Ethical approval was obtained from University of Hail Research Ethics following the rules of the Helsinki Declaration (approval number 26-0150494-2015). Participation was voluntary and no incentives were provided to the participants. Socioeconomic factors included family size, family income, whether the subject’s mother worked, the subject’s mother’s education, and the subject’s father’s education. Lifestyle habits included exercise, watching TV, and sleeping hours. The structured questionnaire that was used in the current study was built upon a previously utilized pre-tested questionnaire developed by Attila and Cakir [14]. However, some modifications were introduced to the questionnaire to be more suitable to the Saudi society regarding the demographic and socioeconomic aspects of life. These modifications were introduced during the process of validating the questionnaire. Body weight of all participants was measured using an electronic scale by trained dieticians and technicians. Height for all the participants was measured by using a stadiometer (Detecto, Webb City, MO, USA). During measurements, participants were wearing light clothes and barefooted.

### 2.3. Data Analysis

Anthroplus software (version 1; http://www.who.int/growthref/tools/en) was used to determine the *Z*-score for students. The ≤−2 SD was used as a cutoff for thinness, which coincides with body mass index (BMI) ≤17 kg/m^2^. The cutoff for overweight was 1–2 standard deviation (SD), which coincides with BMI = 25–30 kg/m^2^, the cutoff for obesity was ≥2 SD, which coincides with BMI ≥30 kg/m^2^, while −2–1 SD was considered as normal, which coincides with BMI = 17–25 kg/m^2^

### 2.4. Statistical Analysis

Missing data (*n* = 26) were not statistically computed, and the data set was cleaned and edited for inconsistencies. Data included frequency and percentages of distributions. An exploratory analysis was performed by SPSS (version 17; IBM Corp., Armonk, NY, USA) to model significant predictors of income, sleeping hours, watching TV, and exercise for their potential associations with BMI because these were the factors identified in previous studies [15,16,17] as potential confounders or as being associated with obesity. Odds ratios (ORs) 95%, confidence interval (CI) 95%, and *β*-coefficient were obtained using the logistic regression model to determine the potential association between some socioeconomic and lifestyle habits with obesity. The tested variables were adjusted for age and any other significant variables. The *p*-values were 2-sided and a *p*-value of <0.05 was considered statistically significant.

## 3. Results

Our results showed that 27.0% of male adolescents in the Hail region were suffering from obesity, while 21.3% of participants were overweight and 5.9% were thinness (Table 2). Results of this study showed that there was a negative association between family size >8 and obesity (OR: 0.68, CI: 0.48–0.92, *p* = 0.05). However, there was no association (*p* > 0.05) between family size with neither thinness nor overweight of adolescent males. In addition, there was no association (*p* > 0.05) between family size of <5 and obesity (Table 3).

Income of <5000 SR (Saudi Riyal) (SR ≈ 0.27 US Dollars) was negatively associated with obesity (OR: 0.59, CI: 0.36–0.97, *p* = 0.03). However, there was no association (*p* > 0.05) between family income with neither overweight nor thinness. In addition, there was no association (*p* > 0.05) between a family income of >5000 SR and obesity (Table 3). 

Our results revealed that whether the subject’s mother worked was negatively associated with thinness (OR: 0.47, CI: 0.23–0.96, *p* = 0.04) and positively associated with obesity (OR: 1.43, CI: 1.03–1.99, *p* = 0.03). Having an educated mother, compared with an illiterate mother, was positively associated with overweight, for mothers who can read and write (OR: 1.93, CI: 1.13–3.3, *p* = 0.02), mothers who had middle school education (OR: 2.30, CI: 1.25–4.25, *p* = 0.01), and mothers who had postsecondary studies (OR: 1.78, CI: 1.11–2.86, *p* = 0.02). Our results showed a positive association between having an educated mother and obesity for mothers who can read and write (OR: 2.20, CI: 1.34–3.6, *p* < 0.01), middle school (OR: 2.18, CI: 1.22–3.9, *p* = 0.01), and postsecondary school (OR: 1.84, CI: 1.18–2.85, *p* < 0.01). However, our results showed that having an educated mother, compared with having an illiterate mother, was not associated (*p* > 0.05) with thinness. In addition, having an educated father did not have an effect on adolescents’ BMI (Table 2).

Our results in Table 4 showed that exercise of >3 times/week and <3 times/week, compared with no exercise, had a negative association with overweight (OR: 0.67, CI: 0.46–0.96, *p* = 0.03, for >3 times/week; OR: 0.44, CI: 0.30–0.63, *p* < 0.01 for <3 times/week). Additionally, exercise, compared with no exercise, showed a negative association with obesity (OR: 0.21, CI: 0.12–0.53, *p* < 0.001, for >3 times/week; OR: 0.42, CI: 0.27–0.66, *p* < 0.001, for >3 times/week). Sleep duration of <6 h, compared with 6–9 h of sleep per day, was shown to have a positive association (OR: 1.20, CI: 1.85–5.95, *p* < 0.01) with obesity. However, there was no association (*p* > 0.05) between sleep duration with either thinness or overweight. Our results did not show an association (*p* > 0.05) between watching TV and BMI.

## 4. Discussion

In this study, we investigated the effect of some socioeconomic factors and lifestyle habits on the prevalence of obesity among adolescent males of the Hail region, Kingdom of Saudi Arabia. The percentages of obesity and overweight among adolescent males were higher than that found by Al-Rukban [6] who studied the prevalence of obesity among adolescent students in the Riyadh region of Saudi Arabia (13.8% and 20.5%, for overweight and obese adolescents, respectively). This is not surprising since another study revealed that the percentage of obesity among adults in the Hail region was the highest in Saudi Arabia [8].

The negative association of family size of >8 with obesity could be due to the limited resources available to the family. Our results of family size could be comparable to that of Amin et al. [18] who found that a family size >6 was negatively associated with obesity for male primary students in the Al-Hassa city of Saudi Arabia (OR: 0.3, CI: 0.2–0.4, *p* < 0.05). However, Mahfouz et al. [7] found no association between family size and BMI in adolescents of the Abha region, Saudi Arabia.

The negative association of family income of <5000 SR with obesity is probably due to the limited food sources available for families suffering from poverty. Our results of family income do not agree with others in Western countries that showed low family income is associated with increased incidence of obesity for adolescents [19,20,21]. The disparity in results is probably due to the fact that, in Western low-income families, the quality of food, rather than the quantity, is limited as in the case of developing countries.

Our results showed that whether the subject’s mother worked is an important factor in the prevalence of both thinness and obesity. The negative association between having a mother who works and thinness could be because whether the subject’s mother worked probably improves the financial status of the families, which would lead to having an amplitude of food available for the family members. Studies have shown that working mothers can lead to less time available for preparing food at home, which leads to consumption of meals prepared at restaurants [22,23]. The Institute of Medicine [24] showed that eating food outside homes will increase the incidence of childhood and adolescent obesity, since food prepared outside home tend to have higher fat density and less nutrient density than food prepared at home [25]. Having a mother who works will lead to less time available for mothers to cook healthy food, and families will then rely on restaurants and fast food. Datar et al. [26] showed that children and adolescents tend to consume more unhealthy food when they have working mothers, and this resulted in positive association with obesity [27,28,29,30].

Interestingly, having an educated mother, compared with an illiterate mother, was shown to have a positive association with both overweight and obesity for all education levels, but not for the secondary school. This is surprising since having an educated mother is assumed to improve family awareness of healthy food choices. Our results could be explained by the fact that mothers with a high education tend to join the workforce and therefore, although this would mean abundance of food for the families, this would be at the expense of quality, since having a working mother, as previously shown, is positively associated with obesity. Having an educated father did not show an effect on the BMI of adolescent male children, as was the case with having an educated mother. This is probably since fathers in the Saudi culture do not play a major role in food choice. Our results of the effect of having an educated father agree with Mahfouz et al. [31]. Our results regarding having an educated mother agree with Al-Saeed et al. [32] but do not agree with Mahfouz et al. [7], who found that having an educated mother did not affect BMI of adolescents. The discrepancies in results might be since we categorized the mothers’ education from illiterate to post-secondary education rather than two categories (illiterate vs. literate) as in the study of Mahfouz et al. [7].

Our results showed that exercise plays a critical rule in influencing BMI of adolescent students in the Hail region, KSA. Exercise was shown to be negatively associated with overweight and obesity. This has been shown in several studies [6,7,32,33]. This showed the importance of exercise for adolescent students in decreasing the prevalence of overweight and obesity.

Our results showed that watching TV had no effect on BMI for adolescents in the Hail region. In addition, other studies showed that there was no effect of watching TV on BMI of adolescents in different regions of Saudi Arabia [6,7,33]. This is probably since students relied on other means of entertainment such as Internet and their mobile phones. Farghaly et al. [33] showed that Saudi students were not occupied with watching TV and computers. These studies likely overshadowed the important factor of cell phones being used among adolescents in Saudi Arabia as a replacement for TV. 

Sleep duration of <6 h/day was positively associated with obesity. Al-Hazzaa et al. [11] showed that normal sleep duration was positively associated with normal BMI for students aged (15–19) in three major cities of Saudi Arabia. Other studies revealed that short durations of sleep was positively associated with obesity in adolescents [34,35,36].

This study is limited by the possible effect of other factors on the prevalence of obesity among adolescent male students of the Hail region, such as use of the Internet and of mobile phones. Other factors were excluded from the study due to the very limited response, such as whether the adolescent lives with both of his parents or with only one of them. 

## 5. Conclusions

This study showed that family size of >8, a family income of <5000 SR, and exercise had a negative association with obesity. Having a mother who works was negatively associated with thinness and positively associated with obesity. Having an educated mother and sleeping >6 h/day were positively associated with obesity. However, this study showed that neither having an educated father nor watching TV affected the BMI of adolescent children in the Hail region, KSA.

## Figures and Tables

**Table 1:** Sociodemographic characteristics of the study group.

Category	
Age (years)	15.8 ± 1.7 *
Family size	8.2 ± 3.1 *
Weight (kg)	66.7 ± 20.4 *
BMI kg/m^2^	24.5 ± 6.9 *
Height (cm)	164.8 ± 9.7 *

* Means ± SD. SD: Standard deviation. BMI: Body mass index.

**Table 2:** Descriptive analysis of data variables.

Category	Frequency	Percentage (%)
Age category		
12–14	498	33.3
15–17	530	39.5
18–19	407	27.2
BMI		
Thinness	88	5.9
Normal	685	45.8
Overweight	318	21.3
Obese	404	27.0
Family Size		
<6	203	13.6
6–8	782	52.3
>8	435	29.1
Family income		
<5000	478	31.9
5001–10,000	414	27.7
10,000–20,000	187	12.5
>20,000	416	27.8
Education of mother		
illiterate	423	28.3
can read	278	18.7
middle school	147	9.8
high school	194	13.0
university	453	30.3
Education of father		
illiterate	135	9.0
can read	313	20.9
middle school	178	11.9
high school	290	19.4
university	579	38.7
Working mother		
Yes	505	33.8
No	990	66.2
Exercise pattern		
>3 times/week	411	27.5
<3 times/week	351	23.5
No exercise	733	49.0
Watching TV		
<1 h/day	212	14.2
1–2 h/day	411	27.5
>2 h/day	69	4.6
No TV	803	53.7
Sleep hours		
<6 h/day	293	19.6
6–9 h/day	854	57.1
>9 h/day	348	23.3

**Table 3:** Potential risks of socioeconomic factors on thinness, overweight, and obesity of adolescent male students *.

BMI Status	Socioeconomic Category	*β*-Coefficient	OR	95% CI	*p*-Value
	**Family Size**				
Thinness	5 or less	−0.86	0.42	0.12–1.44	0.17
	>8	0.27	1.32	0.72–2.38	0.36
	6–8	0	1		
Overweight	5 or less	−0.004	0.99	0.59–1.68	0.99
	>8	−0.02	0.98	0.67–1.43	0.92
	6–8	0	1		
Obese	5 or less	0.05	1.01	0.66–1.67	0.84
	>8	−0.39	0.68	0.48–0.92	0.05
	6–8	0	1		
	**Family income**				
Thinness	<5000 RS	0.28	1.23	0.56–2.80	0.58
	5001–10,000 RS	−0.75	0.47	0.18–1.23	0.13
	10,0001–20,000 RS	−0.71	0.49	0.19–1.31	0.15
	>20,000 RS	0	1		
Overweight	<5000 RS	−0.36	0.70	0.40–1.23	0.22
	5001–10,000 RS	0.27	1.31	0.76–2.26	0.33
	10,0001–20,000 RS	0.21	1.23	0.70–2.14	0.47
	>20,000 RS	0	1		
Obese	<5000 RS	−0.53	0.59	0.36–0.97	0.03
	5001–10,000 RS	0.32	0.73	0.45–1.18	0.20
	10,0001–20,000 RS	0	1.00	0.62–1.63	0.99
	>20,000 RS	0	1		
	**Working mother**				
Thinness	Yes	−0.76	0.47	0.23–0.96	0.04
	No	0	1		
Overweight	Yes	0.05	1.05	0.74–1.52	0.77
	No	0	1		
Obese	Yes	0.36	1.43	1.03–1.99	0.03
	No	0	1		
	**Mother’s education**				
Thinness	Can read and write	0.36	1.43	0.67–3.05	0.36
	Middle school	−1.02	0.36	0.08–1.62	0.18
	Secondary school	0.20	1.22	0.53–2.81	0.64
	Postsecondary school	−0.55	0.58	0.26–1.31	0.19
	Illiterate	0	1		
Overweight	Can read and write	0.66	1.93	1.13–3.30	0.02
	Middle school	0.83	2.30	1.25–4.25	0.01
	Secondary school	0.36	1.39	0.76–2.53	0.29
	Postsecondary school	0.61	1.78	1.11–2.86	0.02
	Illiterate	0	1		
Obese	Can read and write	0.79	2.20	1.34–3.60	<0.01
	Middle school	0.78	2.18	1.22–3.90	0.01
	Secondary school	0.36	1.43	0.82–2.50	0.21
	Postsecondary school	0.61	1.84	1.18–2.85	<0.01
	Illiterate	0	1		
	**Father’s education**				
Thinness	Can read and write	0.26	1.30	0.52–3.24	0.57
	Middle school	−0.58	0.56	0.21–1.45	0.23
	Secondary school	0.48	1.62	0.69–3.79	0.27
	Postsecondary school	−0.77	0.46	0.21–1.05	0.46
	Illiterate	0	1		
Overweight	Can read and write	−0.08	0.93	0.46–1.85	0.82
	Middle school	0.29	1.34	0.78–2.30	0.29
	Secondary school	0.03	1.03	0.53–1.99	0.94
	Postsecondary school	0.21	1.24	0.77–1.99	0.38
	Illiterate	0	1		
Obese	Can read and write	−0.56	0.57	0.29–1.13	0.11
	Middle school	0.07	1.07	0.66–1.75	0.78
	Secondary school	0.1	1.11	0.63–1.96	0.73
	Postsecondary school	−0.05	0.95	0.62–1.45	0.81
	Illiterate	0	1		

* Normal weight is the reference group. Abbreviations: CI: confidence interval; OR: odds ratio.

**Table 4:** Potential risks of lifestyle habits on thinness, overweight, and obesity of adolescent male students *.

BMI Status	Lifestyle Habit	*β*-Coefficient	OR	95% CI	*p*-Value
	**Exercise**				
Thinness	>3 times/week	−0.71	0.49	0.24–1.01	0.06
	<3 times/week	−0.64	0.53	0.27–1.04	0.06
	No	0	1		
Overweight	>3 times/week	−0.41	0.67	0.46–0.96	0.03
	<3 times/week	−0.83	0.44	0.30–0.63	<0.001
	No	0	1		
Obese	>3 times/week	−1.54	0.21	0.12–0.53	<0.001
	<3 times/week	−0.86	0.42	0.27–0.66	<0.001
	No	0	1		
	**Watching TV**				
Thinness	<1 h/day	−0.25	0.78	0.34–1.75	0.55
	1–2 h/day	−0.38	0.69	0.35–1.36	0.38
	>2 h/day	−1.38	0.25	0.03–1.90	0.18
	No TV	0	1		
Overweight	<1 h/day	−0.11	0.89	0.55–1.46	0.65
	1–2 h/day	0.21	1.24	0.85–1.80	0.27
	>2 h/day	−0.1	0.91	0.43–1.89	0.79
	No TV	0	1		
Obese	<1 h/day	0.09	1.09	0.70–1.70	0.70
	1–2 h/day	0.24	1.27	0.89–1.80	0.19
	>2 h/day	0.32	1.37	0.74–2.55	0.32
	No TV	0	1		
	**Sleep Hours**				
Thinness	<6 h/day	−0.34	0.71	0.26–1.94	0.51
	>9 h/day	−0.06	0.95	0.66–1.37	1.08
	6–9 h/day	0	1		
Overweight	<6 h/day	−0.39	0.68	0.45–1.02	0.60
	>9 h/day	0.08	1.08	0.72–1.61	0.70
	6–9 h/day	0	1		
Obese	<6 h/day	1.20	3.32	1.85–5.95	<0.01
	>9 h/day	−0.24	0.79	0.51–1.23	0.29
	6–9 h/day	0	1		

* Normal weight is the reference group.
